# Herbal and Alcohol-Free Mouthwashes as Chlorhexidine Alternatives for Preventing Enamel Demineralization in Orthodontic Patients: An In Vitro Study

**DOI:** 10.3390/dj14030131

**Published:** 2026-02-25

**Authors:** Nyema A. Abualsaud, Shahad T. Alameer, Lama M. Alshamrani, Abdulaziz S. Alamri, Naif N. Almasoud, Suliman Y. Shahin, Mohammed M. Gad, Osama A. Alsulaiman, Abdulrahman A. Balhaddad, Ahmed A. Alsulaiman

**Affiliations:** 1 Department of Preventive Dental Sciences, College of Dentistry, Imam Abdulrahman Bin Faisal University, P.O. Box 1982, Dammam 31441, Saudi Arabia; 2220600006@iau.edu.sa (N.A.A.); 2180000445@iau.edu.sa (S.T.A.); 2190003115@iau.edu.sa (L.M.A.); absalamri@iau.edu.sa (A.S.A.); nnalmasoud@iau.edu.sa (N.N.A.); sshahin@iau.edu.sa (S.Y.S.); oaalsulaiman@iau.edu.sa (O.A.A.); 2 Department of Substitutive Dental Sciences, College of Dentistry, Imam Abdulrahman Bin Faisal University, P.O. Box 1982, Dammam 31441, Saudi Arabia; mmjad@iau.edu.sa; 3 Department of Restorative Dental Sciences, College of Dentistry, Imam Abdulrahman Bin Faisal University, P.O. Box 1982, Dammam 31441, Saudi Arabia; abalhaddad@iau.edu.sa

**Keywords:** aligner, antibacterial mouthwash, bracket, *Streptococcus mutans*, orthodontics

## Abstract

**Background/Objectives:** Chlorhexidine (CHX) and alcoholic (A+) mouthwashes are associated with adverse oral effects. Therefore, this study compared the efficacies of non-alcoholic mouthwashes, including fluoride (A−) and herbal (Hr) rinses, for preventing bacterial accumulation and enamel demineralization around metal brackets (MBs), ceramic brackets (CBs), and resin composite attachments (RCAs). **Methods:** Following the exposure to CHX, A+, A−, and Hr rinses for 1 min, the growth of *Streptococcus mutans* on MB, CB, and RCA was assessed using colony-forming units and scanning electron microscopy (SEM). Controls included attachments without intervention. In another setting, enamel with bonded attachments was exposed to mouthwashes for 1 min and subjected to cariogenic demineralization for 24 h. Enamel’s Vickers microhardness was measured before and after the demineralization challenge. Data were analyzed using paired *t*-tests and one-/two-way ANOVA with Tukey’s tests. **Results:** CHX mouthwash demonstrated superior antimicrobial efficacy against *S. mutans* biofilms across all orthodontic attachments (*p* < 0.05). On metallic brackets, CHX (0 ± 0 log_10_) and A− (1.7 ± 0.4 log_10_) significantly (*p* < 0.001) outperformed controls (6.9 ± 0.1 log_10_), Hr (6.08 ± 0.2 log_10_), and A+ (6.2 ± 0.6 log_10_). Similar patterns emerged for ceramic brackets, with CHX (0 ± 0 log10) and A− (1.4 ± 0 log10) superior to controls (6.6 ± 0.4 log_10_). On resin composite attachments, CHX (2.9 ± 0.05 log_10_) and Hr (3.4 ± 0.08 log_10_) exceeded controls (5.4 ± 0.09 log_10_) in inhibiting the biofilm growth (*p* < 0.05). Enamel microhardness reduction was significantly influenced by attachment type (*p* < 0.0001) and mouthwash type (*p* = 0.0063), with significant interaction between variables (*p* = 0.0052). **Conclusions:** CHX and A− mouthwashes effectively inhibited *S. mutans* biofilms on orthodontic attachments, while attachment type and mouthwash significantly influenced enamel microhardness reduction.

## 1. Introduction

Materials used for orthodontic appliances have undergone several changes, from gold in the 1960s to the stainless steel used today [1]. In the 80s, orthodontic treatment gravitated towards more esthetic options through the introduction of ceramic brackets (CB), and clear aligner therapy has emerged in the 21st century [1]. Thus, several options for orthodontic treatment are currently available [1], which differ not only in esthetics but also in material characteristics, such as surface irregularities and composition and susceptibility to biofilm accumulation around the attachment-enamel interface [2,3].

Along with other factors, poor oral hygiene may cause demineralization of the surrounding enamel [2,3], resulting in porosities in the enamel subsurface and the appearance of white spot lesions. Without intervention, white spot lesions can progress to carious cavitation leading to esthetic concerns [4,5]. Interestingly, the incidence of new lesions among orthodontic patients can reach up to 45.8%, highlighting the need for new approaches to minimize this clinical problem [6].

Orthodontists can prevent potential side effects of increased biofilm accumulation during treatment by providing oral hygiene instructions in the clinic, applying fluoride-containing varnishes, and advising the use of commercially available fluoride-containing dentifrices and mouthrinses [4]. The benefits of fluoride-containing mouthwashes include reduction in white spot lesions by 25% and the potential prevention of enamel demineralization in patients undergoing orthodontic treatment. However, the optimal frequency of use has not been determined [7]. Other mouthwashes that may be prescribed include chlorhexidine (CHX) or alcohol (A+). Although they are effective in preventing demineralization and bacterial growth, their prolonged use is associated with drawbacks, such as dental discoloration and apoptosis and necrosis of human cells caused by CHX [8], and an increased incidence of cancer in patients using A+ mouthwashes, with several studies indicating a statistically significant correlation between frequent use and squamous cell carcinoma of the head and neck [9]. Therefore, investigating other alternative mouthwashes, such as herbal mouthwashes, is critical to advance the oral care with no adverse effects.

Various types of herbal (Hr) mouthwashes, such as *Melaleuca alternifolia* (tea tree oil) and Aloe barbadensis Miller (*Aloe vera*) mouthwashes, are as effective in reducing plaque indices as CHX [10,11]. Aloe vera can reduce Streptococcus mutans growth in patients receiving radiation therapy [12], and tea tree oil has been found to have anti-inflammatory and anti-bacterial effects in both in vitro and in vivo studies; furthermore, it showed a reduction in gingival edema, inflammation, and bleeding comparable to that with CHX [13,14].

Owing to limitations associated with the daily use of CHX and A+ mouthwashes, this study aimed to compare the effectiveness of CHX, A+, A−, and Hr mouthwashes for preventing bacterial accumulation and bacteria-induced demineralization at the orthodontic attachment–enamel interface. The null hypothesis was that there would be no significant differences among CHX, A+, A−, and Hr mouthwashes in their effectiveness for preventing bacterial accumulation and bacteria-induced demineralization at the orthodontic attachment-enamel interface.

## 2. Materials and Methods

### 2.1. Study Design and Sample Size Calculation

**Figure 1:**
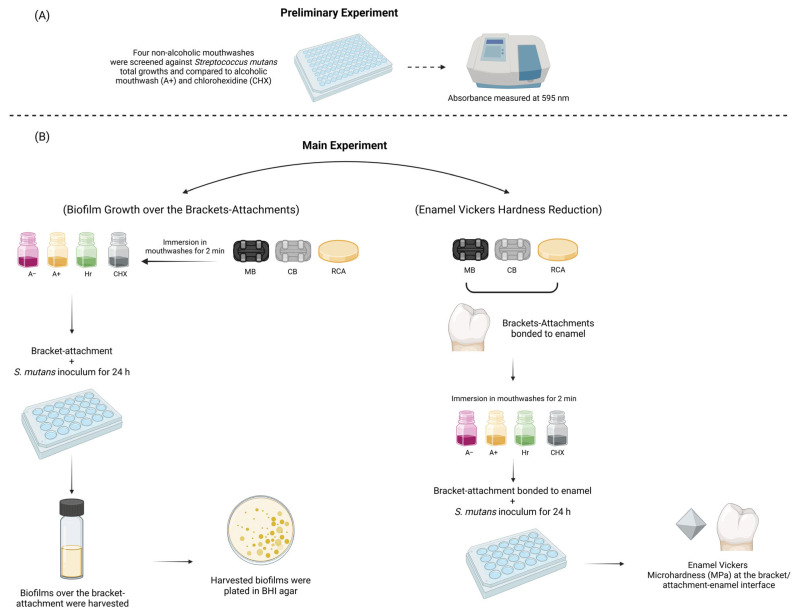
Schematic drawing showing the study design (**A**) preliminary experiment (**B**) main experiment.

This article adheres to the principles outlined in the Declaration of Helsinki. Ethical approval for this study was obtained from the Institutional Review Board of the lmam Abdulrahman Bin Faisal University, (IRB Number: IRB-PGS-2023-02-546; approval date: 6 December 2023). The teeth were not extracted for research purposes, and their use without consent was waived by the IRB (IRB-PGS-2023-02-546) and national regulations. The study design is illustrated in Figure 1. Three types of attachments were investigated: metal bracket (MB), CB, and resin composite attachment (RCA). Before the main experiment, a preliminary experiment was conducted to select the most effective A− mouthwash for comparison with A+ and CHX mouthwashes (Table 1) in the main experiment. Three types of mouthwashes were investigated: A+, A− with fluoride, and Hr. CHX mouthwash and no intervention were used as the positive and negative controls, respectively. This design resulted in a total of 15 groups. A priori sample size calculation was performed using G Power® (v 3.1.9.2., Kiel University, Kiel, Germany)to establish the sample size necessary for a two-way analysis of variance (ANOVA) of the mean scores in the 15 groups at a significance level of *p* < 0.05 [15]. The results indicated a minimum of 18 samples in each group to detect a medium effect size (f = 0.40). Power was set at 85%.

### 2.2. Preliminary Experiment to Select the Most Potent Non-Alcoholic Mouthwashes

The mouthwashes used in the bacterial absorption tests are listed in Table 1. In the preliminary experiment, a single *S. mutans* colony was prepared by defrosting, suspended in 5 mL of brain–heart infusion (BHI) broth (Molequle-On, Auckland, New Zealand), and allowed to grow overnight. On the next day, 96-well plates were inoculated with 142.5 µL of fresh BHI broth supplemented with 2% of sucrose, 10 µL of the overnight *S. mutans* (UA159, American Type Culture Collection, Mannasas, VA, USA) culture, and 47.5 µL of each mouthwash. A group with only BHI with 2% of sucrose and no mouthwash was used as a negative control. While chlorohexidine was used as a positive control. The preliminary experiment was conducted in triplicate, with three samples per group for each replicate, resulting in a total of nine samples. The plates were incubated at 37 °C in an aerobic incubator with 5% CO_2_ for 24 h. After 24 h, the total growth of the bacterial culture was measured using a spectrophotometer (SpectraMax M5, MolecularDevices, Sunnyvale, CA, USA) at 595 nm [16]. This preliminary experiment revealed that the Aloe Dent Hr and Listerine A− mouthwashes were as effective as the A+ mouthwash. Therefore, these two mouthwashes were included in the main experiment, and the other two mouthwashes were excluded.

### 2.3. Effects of Mouthwashes on Biofilm Growth on Orthodontic Attachments

#### 2.3.1. Colony Forming Unit

*S. mutans* colonies were grown overnight in 5 mL BHI. On the next day, MB (Dinamique^®^ self-ligating brackets, DENTAURUM GmbH & Co.KG (Ispringen, Germany)), CB (3M™ Clarity™ Advanced Ceramic Brackets Kit (3M, Saint Paul, MN, USA)), and RCA (Filtek^TM^ Supreme Flowable, 3M, Maplewood, MN, USA) samples were placed in ethanol for 15 min for sterilization; then, they were transferred to 24-well plates containing each mouthwash for 1 min [17]. Thereafter, all attachments were then placed in sterile well plates with 1000 µL fresh BHI broth supplemented with 2% of sucrose and 50 µL of the overnight *S. mutans* culture. After 24 h, the attachments were placed in tubes containing 1 mL of phosphate-buffered saline to harvest the biofilm by sonication and vortexing. The harvested biofilms were diluted and plated onto BHI agar (Molequle-On, Auckland, New Zealand). The plates were incubated for 48 h, and the colonies were counted using a plate counter after considering the dilution factor [18,19]. Groups with no mouthwash treatment were used as the negative controls.

#### 2.3.2. Scanning Electron Microscopy

*S. mutans* biofilm on the orthodontic attachment was prepared as described in Section 2.3.1. The biofilms were rinsed with phosphate-buffered saline and fixed using 3% formaldehyde. The following day, they were dried using serial dilutions of ethanol, followed by 100% hexamethyldisilazane [18]. Finally, the biofilms were sputter coated with platinum and imaged using a scanning electron microscope (SEM; Inspect S50; FEI Company, Brno, Czech Republic). Images were captured at 20 kV using multiple magnifications.

### 2.4. Effect of Mouthwashes on Enamel Demineralization Around the Attachments

#### 2.4.1. Tooth Sample Preparation

Two hundred and seventy enamel samples were prepared from sound teeth collected at Imam Abdulrahman Bin Faisal University Dental Hospital, which produced 18 samples per group. Only teeth with no carious lesions or cracks were selected. The tooth samples were cut to have at least a 5 × 5 mm enamel surface. The specimens were mounted in acrylic resin blocks such that only the enamel surface was visible. The enamel surfaces of all specimens were polished using 600- and 1200-grit silicon carbide papers (Wirtz-Buehler, Düsseldorf, Germany). The samples were subjected to the Vickers microhardness test (Wilson Hardness, ITW Test & Measurement GmbH, Shanghai, China) twice at a load of 300 g for 10 s [20] to standardize the microhardness before starting the experiment and determine the baseline value. Only specimens with comparable microhardness values (±15%) were eligible for the experiment. The microhardness test was done by a single well-trained operator.

#### 2.4.2. Bonding Orthodontic Attachments to the Teeth

For tooth-surface preparation, the enamel surface was etched using 32% phosphoric acid for 30 s, rinsed, and dried [21]. Thereafter, 3M™ Scotchbond™ Universal Adhesive (3M, Maplewood, MN, USA) was applied and cured at full power for 10 s (Satelec Acteon MiniLed active, Acteon, Merignac, France) according to the manufacturer’s instructions [22]. Etching and bonding were restricted to the size of the attachment using a stamping technique by placing the etchant on the attachment surface which is usually in contact with the tooth during bonding, to avoid extending the materials beyond the enamel-attachment interface.

The bonding material was placed using two brushes; one was dipped in the adhesive to add a small drop to the etched surface, and the other was used to rub the adhesive for 20 s to ensure that there was no excess spreading outside the etched borders. This was followed by gentle air drying for 5 s directed away from the unbonded enamel surface and then curing at full power for 10 s [22]. Transbond™ XT Light Cure Adhesive paste (3M, Maplewood, MN, USA) was applied to the bracket surface, the brackets were placed on the tooth surface, and the excess resin was scraped off using a hand scaler. Finally, the resin was cured at full power for 10 s [23]. RCA were bonded using an Invisalign^®^ attachment template [24]. The template was filled with Filtek™ Supreme Flowable (3M, Maplewood, MN, USA) and placed on the tooth surface. Subsequently, the attachment was cured for at least 20 s (Satelec Acteon MiniLed Active, Acteon, Merignac, France) [25].

#### 2.4.3. Effects of Mouthwashes on Enamel Microhardness

*S. mutans* colonies were cultured overnight in 5 mL BHI. The following day, the enamel samples with bonded attachments were soaked in each mouthwash for 1 min and transferred to an empty 24-well plate [17]. Then, 75 µL of the overnight *S. mutans* culture (approximately 10^6^ colony-forming units [CFU]/mL) was added with 1.5 mL of fresh BHI broth supplemented with 2% sucrose [26]. After incubation for 24 h, the attachment-tooth interface was subjected to Vickers microhardness measurements twice. The microhardness values before and after demineralization were compared for each group. In addition, the reduction in microhardness (%) was calculated [27]. All samples were prepared and tested by a single examiner.

### 2.5. Statistical Analysis

Descriptive statistics (means, standard deviations, frequencies, and percentages) were used to summarize the data. Normality was assessed using the Shapiro–Wilk test. One-way ANOVA and Tukey’s multiple comparison tests were used to compare the biofilm growth (CFUs) between the mouthwashes, regardless of the type of attachment. A paired *t*-test was used to compare the enamel microhardness before and after the demineralization challenge. Finally, two-way ANOVA and Tukey’s multiple comparison tests were used to compare the microhardness reduction (%) between the groups and to investigate the interaction between the attachments and mouthwashes used. Data were analyzed using Sigma Plot 12.0 (SYSTAT, Chicago, IL, USA), and statistical significance was set at *p* < 0.05.

## 3. Results

### 3.1. Effects of Selected Mouthwashes on Light Absorption by S. mutans

**Figure 2:**
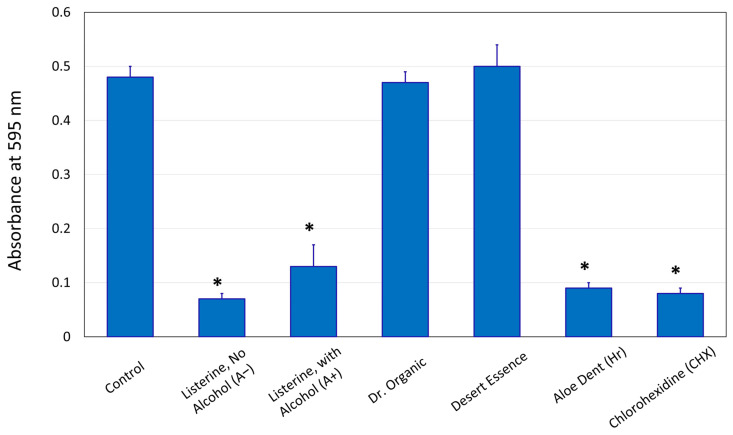
Absorbance values at 595 nm (mean ± SD) for the total growth of *Streptococcus mutans* exposed to different mouthwashes after 24 h incubation. * significantly different than the control at *p* < 0.05.

Figure 2 shows the inhibitory effects of selected mouthwashes on *S. mutans* total growth (*n* = 9 per group). A significant reduction (*p* < 0.05) in *S. mutans* growth was observed after incubation with A− (0.08 ± 0.001), A+ (0.133 ± 0.04), Hr (0.09 ± 0.002), and CHX (0.08 ± 0.002) compared to the control (0.49 ± 0.03). Among the three Hr mouthwashes tested, Aloe Dent (0.09 ± 0.01) showed the lowest light absorption and significant reduction (*p* < 0.05) compared to the control; therefore, it was used in subsequent experiments along with A−, A+, and CHX.

### 3.2. Effects of Mouthwash on S. mutans Accumulation on Orthodontic Attachments

**Figure 3:**
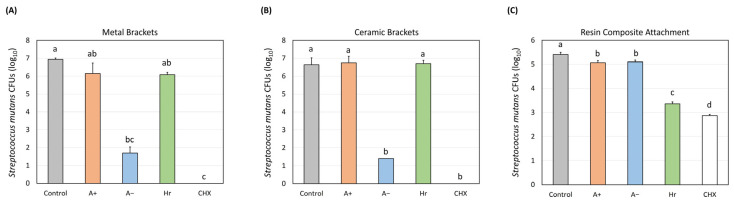
*Streptococcus mutans* biofilm growth on metallic brackets (**A**), ceramic brackets (**B**), and resin composite attachments (**C**) after exposure to different mouthwashes. Values are presented as mean ± standard deviation. Different letters indicate significant differences (*p* < 0.05, one-way analysis of variance).

**Figure 4:**
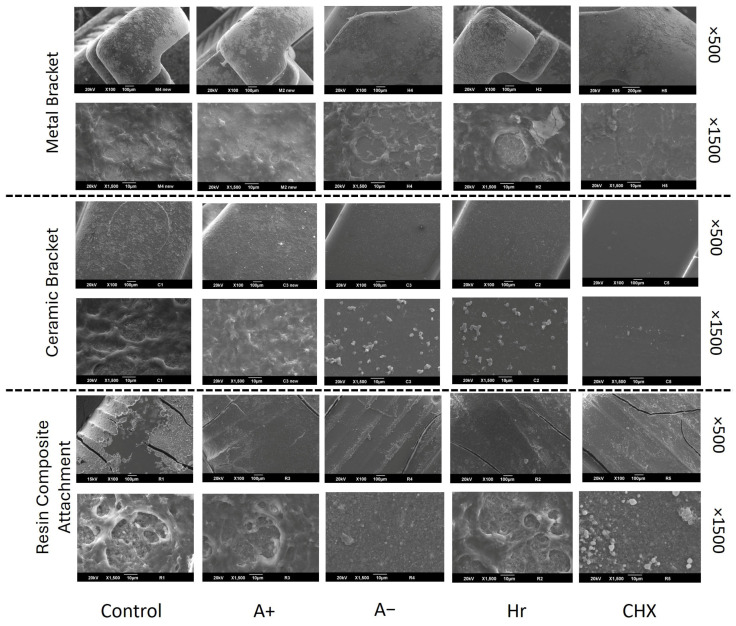
Scanning electron microscope images of the Streptococcus mutans biofilms grown on metallic brackets, ceramic brackets, and resin composite attachments after exposure to different mouthwashes.

CFU log_10_ values for the MB group with *p* < 0.05 are shown in Figure 3A. CHX (0 ± 0 log_10_) had the highest efficacy for *S. mutans* growth inhibition, followed by A− (1.7 ± 0.4 log_10_), which was significantly greater compared to those of the control (6.9 ± 0.1 log_10_), Hr (6.08 ± 0.2 log_10_), and A+ (6.2 ± 0.6 log_10_). The efficacy of CHX and A− were not significantly different (*p* > 0.05). As shown in Figure 3B, CHX (0 ± 0 log_10_) had the highest efficacy for *S. mutans* growth inhibition compared with the control (6.6 ± 0.4 log_10_), Hr (6.7 ± 0.2 log_10_), and A+ (6.8 ± 0.4 log_10_). No significant difference was found between the efficacy of Hr and A+ compared with that of A− (1.4 ± 0 log_10_). Importantly, the efficacy of CHX and A− were not significantly different. Figure 3C shows that CHX (2.9 ± 0.05 log_10_) had the highest efficacy on *S. mutans* growth inhibition compared with the control (5.4 ± 0.09 log_10_), followed by Hr (3.4 ± 0.08 log_10_), A+ (5.1 ± 0.08 log_10_), and A− (5.1 ± 0.08 log_10_). Notably, the efficacy of A− and A+ were not significantly different. Figure 4 shows SEM images of the MB, CB, and RCA groups after the mouthwash experiment. All groups showed changes in surface topography and biofilm accumulation, with less extensive biofilm growth on the specimens exposed to the A− and CHX mouthwashes. Some groups showed the growth of few colonies, for example, on ceramic brackets exposed to CHX, but no growth was reported in the CFUs results. This suggests that the attached colonies had very low metabolic activity and were unable to grow on the agar plates.

### 3.3. Effects of Mouthwashes on the Vickers Hardness of the Enamel

**Figure 5:**
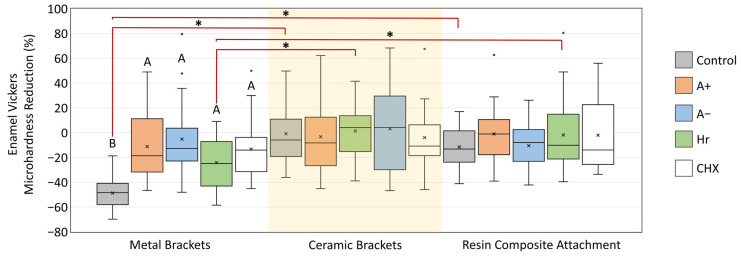
Percentage reduction in the enamel microhardness (at the tooth-attachment interface) after exposure to different mouthwashes followed by the biofilm challenge. Values are presented as mean ± standard deviation. The (*) indicates the significant differences in microhardness at the bracket-attachment interface for each mouthwash type. Different letters indicate significant differences between mouthwashes according to the attachment type. x indicates the mean value.

The paired *t*-test (Table 2) revealed a significant reduction (*p* < 0.05) in the microhardness values (before vs. after) in the MB-control, MB-Hr, MB-CHX, CB-control, and CB-A- groups. No significant reductions were observed in the RCA groups. Table 3 shows the results of the two-way ANOVA for the percentage microhardness reduction; the type of attachment (*p* < 0.0001) and type of mouthwash (*p* = 0.0063) were significant factors associated with enamel microhardness reduction. In addition, a significant interaction (*p* = 0.0052) was observed between the two variables. Figure 5 shows the percentage reduction in the groups. In the MB group, a significant difference was observed between the control and other groups (*p* < 0.05). No significant differences were observed between groups when CB and RCA were used. When the attachments were compared according to the mouthwash type, a significant reduction in microhardness was observed in the MB-control group (*p* < 0.05) compared to the CB-control and RCA-control groups. Similarly, a significant reductio was observed in the MB-Hr group (*p* < 0.05) compared with the CB-Hr and RCA-Hr groups. No other significant differences were observed for the other mouthwashes.

## 4. Discussion

This in vitro study evaluated the effects of chlorhexidine, alcohol-containing, alcohol-free fluoride, and herbal mouthwashes on *Streptococcus mutans* biofilm accumulation and enamel demineralization around different orthodontic attachments. Overall, the findings demonstrated that both mouthwash composition and attachment type influenced bacterial adhesion and enamel microhardness outcomes. Chlorhexidine and the alcohol-free fluoride mouthwash showed greater inhibitory effects on biofilm formation on metallic and ceramic brackets, whereas resin composite attachments exhibited different biofilm behavior and surface changes. In addition, enamel microhardness reduction was significantly affected by attachment type and mouthwash type, highlighting the multifactorial nature of demineralization risk during orthodontic treatment. Mouthwashes containing alcohol may irritate the gingival tissues and cause ulceration and discomfort [28]. In the present study, the ability of A− and Hr mouthwashes was compared to prevent bacterial accumulation and bacteria-induced demineralization at the orthodontic attachment–enamel interface and compared their efficacies with those of A+ and CHX mouthwashes. The results indicated that the null hypothesis was partially rejected, because Hr and A− mouthwashes demonstrated varying degrees of biofilm accumulation and reduced enamel microhardness after bacterial acid challenge.

In the preliminary study, three Hr mouthwash options were tested and compared with the CHX, A+, and A− mouthwashes available over the counter. The results revealed that Aloe Dent was the most effective Hr mouthwash for reducing *S. mutans* growth with efficacy similar to those of the other mouthwashes; therefore, Aloe Dent was used in subsequent experiments. Further investigation revealed that Aloe Dent contains escin, which has anti-inflammatory properties [29]; CI 75810, which has potential antioxidant, anticarcinogenic, and antimutagenic properties [30]; and phenoxyethanol, which is a preservative effective against yeasts and Gram-positive and Gram-negative bacteria that prevents cell growth by inhibiting DNA and RNA biosynthesis as well as causes bacterial cell death [31,32].

The results of the CFU experiment revealed that in the MB and CB groups, CHX demonstrated the highest *S. mutans* inhibition, followed by the A− mouthwash from Listerine, with no statistical difference between the efficacies of the CHX and A− mouthwashes. Investigation of the ingredients of the A− mouthwash revealed that it contained sodium fluoride (220 ppm). Our findings are consistent with those of other studies comparing CHX and fluoride in reducing *S. mutans* activity [33,34]. However, the results for the RCA group showed a lower effectiveness of CHX and Hr in inhibiting *S. mutans* growth compared to both bracket groups. In contrast, bacterial growth reduction in the RCA group was not significantly different between the A+ and A− mouthwashes. This could be due to differences in material composition or surface irregularities in RCA. Further studies are required to compare the effects of mouthwashes according to the characteristics of different resins used in clear aligner therapy, such as surface irregularities and material composition.

SEM revealed varying levels of bacterial adhesion among the MB, CB, and RCA groups. The topographic changes in the RCA group were the most evident, as shown in Figure 4. This may explain the differences in CFU results among the RCA, MB, and CB groups. There were some discrepancies between the CFU and SEM results; some groups with no growth on culture (Figure 3) showed colony formation under SEM (Figure 4). This may be due to the low metabolic activity of these colonies. Agar plates occasionally do not allow the growth of all live colonies, and colonies with low metabolic activity may not be able to grow on agar plates [35]. Although previous studies have evaluated orthodontic aligners [36], evidence regarding biofilm accumulation and topographic changes in the RCA in the oral cavity is limited. The present in vitro study provides an initial understanding of the effect of mouthwashes on resin composite attachments used in clear aligner therapy. However, the long-term effects of mouthwash use on RCA in the oral environment were not evaluated.

The Vickers microhardness test evaluates the hardness of a substrate by applying a specific load to a diamond indenter that creates a small indentation [37,38]. The dimensions of the indentation correspond to the hardness of the material. Vickers microhardness is an effective parameter for evaluating enamel demineralization, because it provides insights into the physical integrity and mineral content of the enamel [37]. A decrease in the Vickers microhardness suggests a loss of mineral content [37]. This process often occurs because of the acidogenic activity of the bacteria grown on the enamel, causing hydroxyapatite crystal dissolution. By assessing the microhardness before and after exposure to the demineralizing challenge, either chemical or microbial as in this study, the extent of enamel loss can be quantified [37,38].

The microhardness results showed that the MB group displayed the most significant reduction in enamel microhardness compared with the CB and RCA groups, and the reduction in microhardness was not significantly different between the CB and RCA groups. These findings may be attributed to the higher biofilm values of MB [39]. Regarding the effect of different types of mouthwash on the enamel in the MB group, CHX showed the least microhardness reduction, followed by the A+ mouthwash, and finally the A− mouthwash. These findings differ from those in a previous study, which reported that fluoride-containing mouthwashes showed less microhardness reduction than those containing CHX [40]. This could be attributed to the duration required for the fluoride in the A− mouthwash to affect the enamel microhardness [7]. SEM images revealed that in this study, RCA showed more topographic changes compared with MB and CB. Additionally, the CFU results showed an inferior ability of the mouthwash to reduce biofilm accumulation in the RCA group than in the MB and CB groups. However, these findings were not consistent with a lower microhardness reduction around RCA compared with that around MB. This may indicate that MB biofilm values, as reported in previous studies, has a greater impact on enamel microhardness reduction [39]. Further studies are required to determine the long-term effects and in vivo performance of the mouthwashes included in this study.

The results of this study emphasize the critical role of mouthwashes in oral hygiene, complementing traditional practices such as brushing and flossing to prevent dental caries. While brushing eliminates plaque and food elements from the surfaces of teeth and flossing aids cleaning between them, mouthwashes deliver an additional layer of defense by delivering bioactive ingredients that can reduce the bacterial load, reinforce the enamel, and counterbalance acids [41,42]. Additionally, mouthwashes can reach areas that are inaccessible to brushing and flossing, thereby enhancing overall oral health [41,42]. Integrating mouthwashes into daily dental-care practices can significantly lower the risk of caries, especially in patients undergoing orthodontic treatment, in whom cleaning is more critical than in regular patients.

The present study had some limitations. First, this study did not compare the effects of the tested mouthwashes in a clinical setting. Second, the experiment only included *S. mutans* and did not consider the complex bacterial microbiota found in the oral cavity and the effects of individual oral-hygiene or dietary habits. Additionally, the differences between the CFU counts and microhardness results could be further investigated. Finally, only two bracket brands and one type of resin were investigated. Future investigations should focus on other brands, as chemical composition can play a significant role in microbial adhesion to substrates. Future research should focus on validating these findings under more clinically relevant conditions. In particular, in vivo and longitudinal studies are needed to assess the sustained effects of herbal and alcohol-free fluoride mouthwashes on biofilm accumulation and enamel demineralization during orthodontic treatment. Further investigations should also evaluate the influence of saliva, dietary challenges, and multispecies oral biofilms, as well as compare different resin materials and bracket designs, as surface characteristics may play a critical role in bacterial adhesion. From a practical perspective, the present findings suggest that mouthwash composition and attachment material should be considered when selecting adjunctive oral hygiene measures for orthodontic patients; however, these implications remain limited to controlled experimental conditions and should not be extrapolated to clinical practice without further evidence.

## 5. Conclusions

Within the limitations of this in vitro study, mouthwash composition and orthodontic attachment type influenced Streptococcus mutans biofilm formation and enamel microhardness following a cariogenic challenge. Chlorhexidine and the alcohol-free fluoride mouthwash demonstrated greater inhibitory effects on biofilm accumulation on metallic and ceramic brackets, whereas the herbal mouthwash showed modest antimicrobial effects on resin composite attachments.

Importantly, these findings are limited to controlled laboratory conditions and do not account for the complex biological, behavioral, and environmental factors present in the oral cavity. Therefore, in vivo and long-term longitudinal studies are required to validate these results, assess sustained antimicrobial efficacy, and evaluate potential adverse effects associated with prolonged mouthwash use. Future research should also investigate patient-related factors, material variability, and real-world oral hygiene practices before any clinical recommendations can be established.

## Figures and Tables

**Table 1:** List of mouthwashes used in the preliminary and main experiments.

Type of Mouthwash	Manufacturer/Distributer	Composition	Exclusion/Inclusion from the Main Experiment
Herbal (Organic Tea Tree, Arnica, Aloe Vera and grapefruit seed extract)	(Dr.Organic, Dr.Organic Ltd., Swansea, UK)	Aloe barbadensis leaf juice, Aqua, Sorbitol, Polysorbate, Glycerin, Pyrus malus (Apple) fruit extract, Cetraria islandica (Icelandic moss) extract, Citrus grandis (Grapefruit) extract, Centella asiatica (Gotu kola) extract, Melaleuca alternifolia (Tea tree) leaf oil, Sodium lauroyl sarcosinate, Aroma, Menthol, Xylitol, Arnica montana flower extract, Sodium hydroxymethylglycinate, Sodium benzoate, Potassium sorbate, Citric acid, Citral, Linalool, Limonene.	Excluded
Herbal (Tea Tree Oil)	(Desert Essence, Hauppauge, NY, USA)	Water (aqua), glycerin (plant derived), polysorbate 80, Eco-Harvest^®^ melaleuca alternifolia (tea tree) leaf oil, aloe barbadensis leaf juice, mentha viridis (spearmint) leaf oil, hamamelis virginiana (witch hazel) extract, ascorbic acid (vitamin C), calcium ascorbate, citric acid.	Excluded
Herbal (aloe barbadense miller and melaleuca alternifolia)	(AloeDent, Optima Consumer Health, Swansea, UK)	Aqua, Aloe Barbadensis (Aloe Vera) Leaf Juice, Sorbitol, Polysorbate 20, Citrus Grandis (Grapefruit) Seed Extract, Mentha Piperita (Peppermint) Oil, Sodium Lauroyl Sarcosinate, Aroma, Menthol, Melaleuca Alternifolia (Tea Tree) Leaf Oil, Escin, Centella Asiatica (Gotu Kola) Flower/Leaf/Stem Extract, Xylitol, Phenoxyethanol, Citric Acid, CI 75810 (Chlorophyllin-Copper Complex), Limonene, Linalool.	Included
Chlorhexidine Gluconate	(AVOHEX Mouth Wash, Avalon Pharma, Middle East Pharmaceutical Industries Co, Ltd., Riyadh, Saudi Arabia)	Chlorhexidine Gluconate 0.2% *w*/*v*.	Included
Alcohol containing mouthwash (21.6%)	Listerine Italy Johnson & Johnson Gmbh, D-41470 Neuss, DE, UAE.	Aqua, Alcohol, Sorbitol, Poloxamer 407, Eucalyptol, Benzoic Acid, Sodium Benzoate, Methyl Salicylate, Thymol, Sodium Saccharin, Menthol, Aroma, CI 42053.	Included
Non-alcohol containing mouthwash (Sodium Fluoride 220 ppm)	(Listerine, Johnson & Johnson Gmbh, Riyadh, Saudi Arabia)	Aqua, Propylene Glycol, Sorbitol, Poloxamer 407, Sodium Lauryl Sulfate, Eucalyptol, Benzoic Acid, Sodium Benzoate, Methyl Salicylate, Thymol, Sodium Saccharin, Sodium Fluoride, Menthol, Sucralose, Aroma, CI 42053. Contains Sodium Fluoride (220 ppm F^−^).	Included

**Table 2:** Microhardness values of the enamel with bonded orthodontic attachments before and after the bacterial demineralization challenge.

	Metal-Control		Metal-Herbal		Metal-A+		Metal-A−		Metal-CHX	
	Before	After	Before	After	Before	After	Before	After	Before	After
**Mean**	501.808	254.41 *	486.016	369.822 *	494.358	430	471.083	425.275	470.18	389.808 *
**SD**	74.822	55.399	86.83	98.971	86.293	119.589	104.468	93.803	106.972	75.258
	Resin-Control		Resin-Herbal		Resin-A+		Resin-A−		Resin-CHX	
	Before	After	Before	After	Before	After	Before	After	Before	After
**Mean**	530.191	511.344	511.016	498.755	509.730	476.061	548.222	538.863	574.963	537.641
**SD**	86.8	93.456	82.536	117.52	101.383	103.885	110.258	80.059	92.287	99.106
	Ceramic-Control		Ceramic-Herbal		Ceramic-A+		Ceramic-A−		Ceramic-CHX	
	Before	After	Before	After	Before	After	Before	After	Before	After
**Mean**	522.5	450.680 *	503.947	467.986	500.938	473.855	553.433	481.016 *	491.327	461.136
**SD**	85.06	63.458	122.454	84.446	104.917	50.409	107.282	74.255	101.73	78.09

Values are presented as mean ± standard deviation. A (*) indicates significant difference (*p* < 0.05, paired *t*-test) between pre- and post-treatment values.

**Table 3:** Two-way analysis of variance to evaluate the effect of mouthwash type, attachment type, and their interactions on enamel microhardness after the biofilm challenge.

	DF	SS	MS	F	*p*
Attachment Type	2	18,039.92	9019.96	14.34	<0.0001
Mouthwash Type	4	9224.71	2306.17	3.67	0.0063
Attachment Type * Mouthwash Type	8	14,189.53	1773.69	2.82	0.0052
Residual	255	160,373.29	628.91		
Total	269	201,827.47			

(*p* < 0.05, Two-way ANOVA). * Interaction between attachment type and mouthwash type (two-way ANOVA).

## Data Availability

The original contributions presented in this study are included in the article. Further inquiries can be directed to the corresponding author.

## Abbreviations

The following abbreviations are used in this manuscript:

A−	(A−)Fluoride Mouthwash
Hr	(Hr)Herbal Mouthwash
MB	(MB)Metal bracket
CM	(CM)Ceramic bracket
RCA	resin composite attachment
CHX	Chlorhexidine
A+	Alcoholic Mouthwash
